# Editorial: Systemic sclerosis: immunological impacts

**DOI:** 10.3389/fimmu.2026.1814196

**Published:** 2026-03-05

**Authors:** Kazuhiro Komura, Takashi Matsushita

**Affiliations:** 1 Department of Dermatology, Kanazawa Red Cross Hospital, Japanese Red Cross Society, Kanazawa, Japan; 2 Department of Dermatology, Kanazawa University Graduate School of Medical Sciences, Kanazawa, Japan

**Keywords:** convergence, framework & architecture, precision medicine, systemic scleroderma, systemic scleroderma (PSS)

Systemic sclerosis (SSc) is characterized by immune activation, vascular injury, and progressive fibrosis. Despite advances in targeted therapies, its marked clinical heterogeneity continues to challenge mechanistic interpretation and therapeutic precision. The six studies assembled in this Research Topic suggest that SSc can be understood as a layered regulatory architecture, in which serological structure, molecular convergence, vascular remodeling, and therapeutic modulation intersect along identifiable axes of control.

Komura reviews the clinical utility and standardization of SSc-related autoantibodies, emphasizing that ACA, ATA, and RNAP III are not merely diagnostic markers but define organ-risk trajectories and disease evolution across populations. A harmonized workflow (ANA/ICAP followed by core ELISAs and reflex assays) provides a reproducible framework for stratifying heterogeneity, establishing serology as a structural baseline for downstream molecular and clinical interpretation.

At the molecular level, Wu et al. identify NOX4 and NEK6 as shared elements linking SSc and sarcopenia through integrative transcriptomics and machine learning. Their findings highlight convergence within oxidative–immune regulatory networks beneath divergent clinical phenotypes, suggesting that heterogeneous manifestations may arise from intersecting molecular control points rather than isolated pathogenic cascades.

Napolitano et al. demonstrate fibrinolytic–vascular imbalance in SSc, characterized by reduced free uPA and elevated PAI-1, with PAI-1 levels correlating with capillaroscopic loop shortening. This directly connects circulating biochemical alterations to structural microvascular remodeling. Their comparison of suPAR assay platforms further underscores that biological interpretation depends on molecular form detection, bridging serum profiling with tissue architecture.

Moving toward regulatory restoration, Lee et al. show that lipid nanoparticle–mediated delivery of A20 mRNA suppresses TRAF6/NF-κB signaling and attenuates fibrosis *in vivo*. Rather than targeting a single cytokine, this strategy recalibrates a central regulatory brake within the immune–fibrotic network, illustrating how pathway-level modulation can exert broad downstream influence.

Clinical translation of layered intervention is exemplified by Fu et al., who report stabilization of severe cardiac involvement using sequential cyclophosphamide followed by tocilizumab. This phased modulation of immune axes demonstrates that multi-level therapeutic strategies can reverse life-threatening organ manifestations, supporting the concept of systemic recalibration through coordinated intervention.

Finally, recent comprehensive analyses of SSc pathogenesis emphasize the intricate interplay among immune activation, cytokine networks, TGF-β–driven fibroblast signaling, and extracellular matrix remodeling (Jimenez et al.). This multidimensional framework underscores the density and redundancy of immune–fibrotic interactions in SSc. In such a networked context, therapeutic impact may depend less on suppressing isolated mediators and more on understanding where coordinated regulatory influence can propagate across layers.

**Figure 1:**
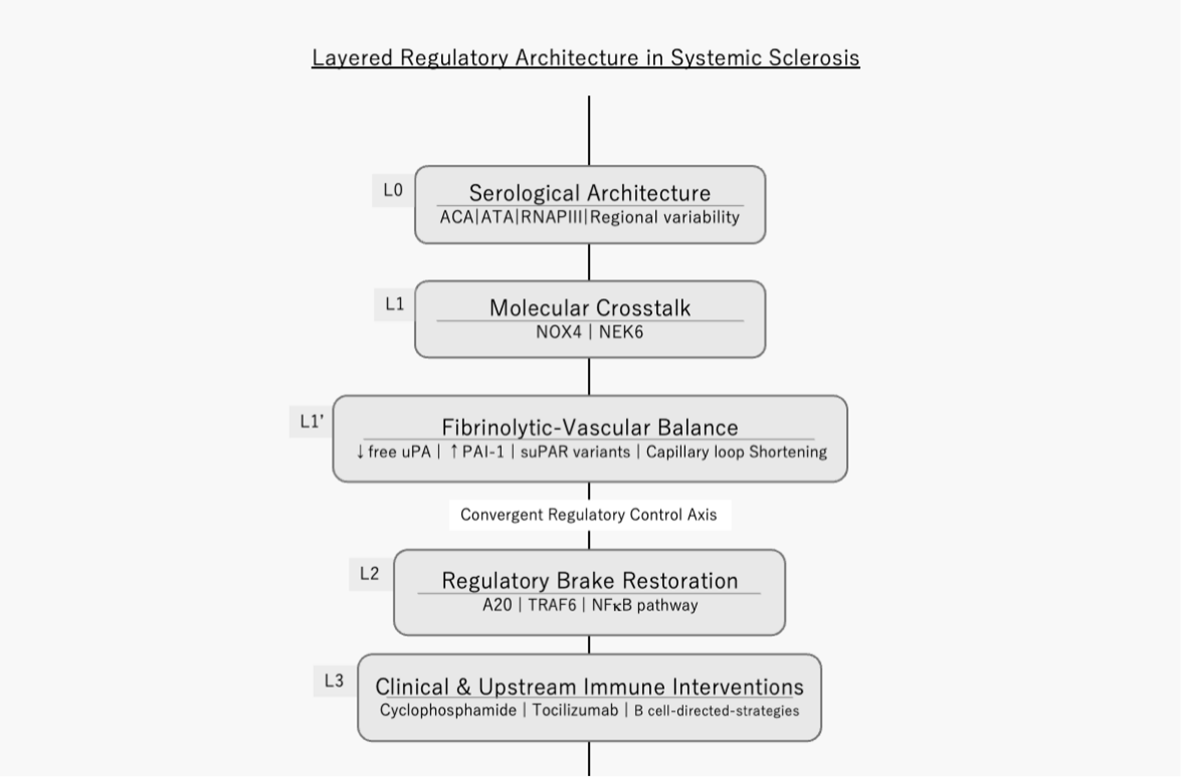
Layered regulatory architecture and convergent control in systemic sclerosis. Systemic sclerosis (SSc) is represented as a vertically organized regulatory structure composed of interacting layers. L0 (Serological Architecture) defines baseline trajectories through ACA, ATA, RNAP III, and regional variability. L1 (Molecular Crosstalk) highlights convergence nodes such as NOX4 and NEK6. L1′ (Fibrinolytic–Vascular Balance) links reduced free uPA, elevated PAI-1, altered suPAR variants, and capillary loop shortening. The central vertical axis denotes convergent regulatory control across layers. L2 illustrates regulatory brake restoration via A20–TRAF6–NF-κB signaling. L3 captures clinical and upstream immune interventions, including cyclophosphamide, tocilizumab, and B-cell-directed strategies. Together, the model emphasizes structural convergence across immune, vascular, and fibrotic domains.

Collectively, these studies define a vertically organized regulatory architecture encompassing serological structure, molecular convergence, fibrinolytic–vascular balance, regulatory brake restoration, clinical layered intervention, and upstream immune modulation. Although SSc appears clinically heterogeneous, structural analysis reveals points where multiple regulatory processes intersect. These are not necessarily the most abundant mediators but loci where distinct layers converge. This vertically organized regulatory model is illustrated in **Figure 1**.

Rather than expanding catalogs of dysregulated molecules, future progress may depend on clarifying how regulatory layers interact and where convergence occurs. A structurally informed perspective may therefore support more precise and reproducible therapeutic strategies in complex autoimmune disease.

